# Dissociation of eIF4E-Binding Protein 2 (4E-BP2) from eIF4E Independent of Thr^37^/Thr^46^ Phosphorylation in the Ischemic Stress Response

**DOI:** 10.1371/journal.pone.0121958

**Published:** 2015-03-30

**Authors:** María I. Ayuso, Emma Martinez-Alonso, Nelida Salvador, Petra Bonova, Ignacio Regidor, Alberto Alcázar

**Affiliations:** 1 Department of Investigation, Hospital Ramón y Cajal, IRYCIS, Madrid, Spain; 2 Institute of Neurobiology, Slovak Academy of Sciences, Košice, Slovakia; 3 Department of Neurophysiology, Hospital Ramón y Cajal, IRYCIS, Madrid, Spain; University of British Columbia, CANADA

## Abstract

Eukaryotic initiation factor (eIF) 4E-binding proteins (4E-BPs) are translational repressors that bind specifically to eIF4E and are critical in the control of protein translation. 4E-BP2 is the predominant 4E-BP expressed in the brain, but their role is not well known. Here, we characterized four forms of 4E-BP2 detected by two-dimensional gel electrophoresis (2-DGE) in brain. The form with highest electrophoretic mobility was the main form susceptible to phosphorylation at Thr^37^/Thr^46^ sites, phosphorylation that was detected in acidic spots. Cerebral ischemia and subsequent reperfusion induced dephosphorylation and phosphorylation of 4E-BP2 at Thr^37^/Thr^46^, respectively. The induced phosphorylation was in parallel with the release of 4E-BP2 from eIF4E, although two of the phosphorylated 4E-BP2 forms were bound to eIF4E. Upon long-term reperfusion, there was a decrease in the binding of 4E-BP2 to eIF4E in cerebral cortex, demonstrated by cap binding assays and 4E-BP2-immunoprecipitation experiments. The release of 4E-BP2 from eIF4E was without changes in 4E-BP2 phosphorylation or other post-translational modification recognized by 2-DGE. These findings demonstrated specific changes in 4E-BP2/eIF4E association dependent and independent of 4E-BP2 phosphorylation. The last result supports the notion that phosphorylation may not be the uniquely regulation for the binding of 4E-BP2 to eIF4E under ischemic stress.

## Introduction

Ischemia induces a period of hypoxia and energy depletion inducing an inhibition of translational rates [1]. Normal oxygen and energy levels can be restored in the subsequent reperfusion period upon reoxygenation(White et al. 2000). However, the initial period of reperfusion increases reactive oxygen species production and causes additional stress [2]. The ischemia and ischemia-reperfusion (IR) affects different tissues, being the brain particularly sensitive to these stresses, where the restoration of translation inhibition is delayed compared with that of energy metabolism or ion homeostasis [3].

The translation process has an important control point in the recruitment of the 40S ribosomal subunit to the 5' end of mRNA. In this process, a key step is the assembly of eukaryotic initiation factor (eIF) 4F complex, which contains eIF4A, an ATP-dependent RNA helicase; eIF4E, which binds to the mRNA 5'-cap structure m^7^GpppN (7-methylguanosine triphosphate, where N is any nucleotide); and eIF4G, a scaffolding protein that provides docking sites for the aforementioned initiation factors [4]. eIF4E recruits eIF4G and eIF4A to assemble the eIF4F complex and bind to the 5' cap [5]. The activity and/or availability of eIF4E are a limiting step in translation initiation and are a primary factor in the control of gene expression. The translational repressors named eIF4E-binding proteins (4E-BPs)—that in mammals comprises three members (4E-BP1, 4E-BP2 and 4E-BP3)—bind to eIF4E, compete with eIF4G and inhibit eIF4G binding to eIF4E, which prevents eIF4F complex formation and inhibits cap-dependent translation [5, 6]. The best characterized 4E-BP protein, 4E-BP1, is one of the main effectors of mTOR, a serine/threonine-protein kinase in the phosphoinositide 3-kinase (PI3K)-protein kinase B (PKB or Akt) signaling pathway that integrates signals from extracellular stimuli, amino acid availability, and the oxygen and energy status of the cells [6, 7]. The (hyper)phosphorylation of 4E-BP1 reduces its affinity for eIF4E, allows eIF4E to bind eIF4G to form the eIF4F complex and the cap-dependent translation. Conversely, the (hypo)dephosphorylated forms of 4E-BP1 bind to eIF4E, which leads to translation inhibition [5–7]. Phosphorylation of Thr^37^/Thr^46^ on 4E-BP1 appears to be the critical step because it is the priming event for subsequent (hyper)phosphorylation and prevention of the binding to eIF4E in cell lines [6], and regulates the binding to eIF4E in brain tissue [8].

Previously, we have reported dephosphorylation of 4E-BP1 after ischemia and induction of 4E-BP1 phosphorylation during ischemic reperfusion [8]. These changes in 4E-BP1 phosphorylation in IR agree with those found for PKB and mTOR kinase activities [8]. However, the lack of knowledge about 4E-BP2 regulation led us to investigate the phosphorylation regulation of 4E-BP2 in brain, where 4E-BP2 is highly expressed [9]. In the present report we study the phosphorylation and isoforms of 4E-BP2 and its association to eIF4E induced by IR stress with short- and long-term reperfusion in the cerebral cortex and the hippocampal *cornu ammonis* 1 (CA1) region. Cerebral cortex and CA1 were used as resistant and vulnerable regions, respectively, against global cerebral ischemia [10, 11]. In addition, we discovered specific changes in the association of 4E-BP2 to eIF4E, demonstrating that the dissociation of 4E-BP2 and eIF4E can be independent of 4E-BP2 phosphorylation.

## Materials and Methods

### Materials

Rabbit polyclonal anti-4E-BP2 (#2845), anti-phospho-4E-BP1/2 (#9459) (Thr^37^ and/or Thr^46^ of the human sequence) and anti-phospho-ribosomal protein S6 (rpS6) (#4858) (Ser^235^/Ser^236^) antibodies were from Cell Signaling. Mouse monoclonal anti-eIF4E antibody (610269) was from BD Transduction Labs and anti-rpS6 antibody (#2317) was from Cell Signaling. Goat polyclonal anti-eIF4G antibody (n-20, sc-9601) was from Santa Cruz Biotechnology. Rabbit polyclonal anti-β-tubulin antibody (PRB-435P) was from Covance. The chemicals used in isoelectric focusing and SDS-PAGE were purchased from Bio-Rad and GE Healthcare. All general chemicals were purchased from Sigma unless stated otherwise.

### Animal model of ischemia and IR

Transient global forebrain ischemia was induced in adult male Wistar rats (10–12 weeks, from Charles River) by the standard four-vessel occlusion model described previously [12, 13]. Briefly, both vertebral arteries were irreversibly occluded by electrocoagulation under anesthesia with a mixture of atropine, ketamine and diazepam (0.25, 62.5, and 5 mg/kg, respectively) delivered by intra-peritoneal injection. After 24 h, both common carotid arteries were occluded for 15 min to induce ischemia and then the animals were sacrificed (I15 group). For IR, animals underwent 15 min ischemia, both clips were removed from the carotid arteries for 30 min- or 3 day-reperfusion (R30 and R3d, respectively), and the animals were sacrificed. Sham control (SHC and SHC3d) animals were prepared in the same way as the R30 and R3d animals, respectively, but without carotid occlusion. All procedures associated with animal experiments were approved by The Ethics Committee of the Hospital Ramon y Cajal, Madrid, Spain, and in accordance with the ARRIVE (Animal Research: Reporting In Vivo Experiments) guidelines.

### Sample preparation

The cerebral cortex and hippocampal CA1 region from control, ischemic and IR animals were rapidly dissected out under a magnifying glass. The samples were homogenized 1:5 (w/v) with buffer A (20 mM Tris-HCl, pH 7.5; 140 mM potassium chloride; 5 mM magnesium acetate; 1 mM dithiothreitol; 2 mM benzamidine; 1 mM EDTA; 2 mM EGTA; 10 μg/ml pepstatin A, leupeptin and antipain; 20 mM sodium β-glycerophosphate; 20 mM sodium molybdate; 0.2 mM sodium orthovanadate), as described previously [12, 14]. The homogenate was centrifuged at 11000 × g for 15 min to obtain a postmitochondrial supernatant (PMS). All procedures were performed at 4°C. The PMS fraction corresponding to each animal was separately kept at -80°C until used and protein concentrations were determined for each sample.

### Binding of 4E-BP2 and eIF4G to eIF4E

In order to study eIF4F complex formation and the binding of 4E-BP2 and eIF4G to eIF4E, a cap-containing matrix—7-methyl-GTP (m^7^GTP)-Sepharose—was used [15] as described previously [8, 16]. PMS samples (300 μg) for each experimental condition were added to m^7^GTP-Sepharose 4B (GE Healthcare; 30 μl of 50/50 w/v slurry) and incubated for 30 min at 4°C in buffer A with 100 mM potassium chloride and 100 μM GTP. The beads were centrifuged at 2500 × *g* for 5 min and washed in the same buffer three times. The proteins were removed from m^7^GTP-Sepharose with SDS loading buffer and subjected to SDS-PAGE or two-dimensional gel electrophoresis and western blotting. The immunoblots were developed separately with the antibodies described above against eIF4G, eIF4E and 4E-BP2 and quantified as described below.

### 4E-BP2 immunoprecipitation

PMS samples (300 μg) of each experimental condition were incubated with 2 μg of rabbit polyclonal anti-4E-BP2 antibody (Sigma) overnight and then further incubated with Protein G-Agarose 4 (ABT) for 1 h at 4°C on rotating shaker. Immunoprecipitated proteins were then washed 3 times in lysis buffer and subsequently subjected to SDS-PAGE or two-dimensional gel electrophoresis and western blotting.

### Western blot analysis

Samples of PMS (35 μg), m^7^GTP-Sepharose or 4E-BP2 immunoprecipitates of each different experimental condition were analyzed by SDS-PAGE (7.5% acrylamide for eIF4G and eIF4E and 15% acrylamide for 4E-BP2; 3% cross-linking) (GE Healthcare) or two-dimensional gel electrophoresis (see below) and transferred onto PVDF membranes (GE Healthcare). The membranes were incubated for 1 h at room temperature or overnight at 4°C with the antibody against the specific protein to be detected, washed, then incubated for 1 h with peroxidase-conjugated anti-mouse,-rabbit (both from GE Healthcare) or-goat (Santa Cruz Biotechnology) IgG, and developed with ECL reagent (GE Healthcare). For phospho-protein detection, the blots were probed with the phospho-specific antibody, stripped, and reprobed with the corresponding anti-total protein antibody. Phospho-4E-BP2 and 4E-BP2 were also analyzed in twin separate experiments to avoid the potential cross-reaction between the different rabbit polyclonal antibodies. The western blots were quantified using ImageQuant TL software (GE Healthcare). Internal standards (tubulin) were included to normalize the different immunoblots. Data of the phospho-forms or phospho-proteins were expressed in arbitrary units with respect to the levels of total protein (ratios). Protein markers (range: 12–225 kDa) (GE Healthcare) were used to calculate the apparent molecular weight (MW).

### Two-dimensional gel electrophoresis (2-DGE)

Samples of PMS (75 μg) or m^7^GTP-Sepharose of each experimental condition were added to 8.5 M urea/5% β-mercaptoethanol (Bio-Rad) and loaded into horizontal IEF slab gels as the first dimension. IEF was performed with immobilized pH 3‒10 nonlinear gradient (3% v/v Bio-Lyte 4/6, 2% v/v Bio-Lyte 3/10) (Bio-Rad) strips (10 cm) in a flatbed Multiphor II Electrophoresis System (GE Healthcare), according to the manufacturer’s instructions. The first dimension was combined with standard vertical slab SDS-PAGE as the second dimension (12% acrylamide; 2.6% cross-linking) (GE Healthcare) performed in 1.0 mm thick gels with the IEF strip used as stacking gel, as described previously [12, 17, 18]; and then proteins identified by western blot. Protein markers (see above) and pI standards (range: 3–10) (GE Healthcare) were used to calculate the apparent MW and pI of identified proteins.

In other experiments, immunoprecipitated samples were labeled on their lysine residues with Cy3 and Cy5 minimal dyes (GE Healthcare). Pairs of experimental and control samples were mixed and analyzed by 2-DGE as described above. The fluorescent gels were scanned using a Typhoon 9200 imager (GE Healthcare).

### Statistical analysis

The different animals from each experimental condition were independently analyzed in duplicate and their averaged values were used for statistical analysis. Data were represented in arbitrary units and expressed as mean ± SEM of the 4–8 different animals. Statistical analysis was performed either using ANOVA following Dunnett’s post-test, when was significant, or by Student’s *t* test to compare the data respect to the SHC or SHC3d control groups. Comparisons between the cerebral cortex and hippocampal CA1 regions were performed using Newman-Keuls post-test after ANOVA, when was significant, or by paired *t* test. Statistical significance was set at p < 0.05 using Prism statistical software (GraphPad Software).

## Results

### IR stress induces phosphorylation of 4E-BP2 at Thr^37^/Thr^46^ sites

**Fig 1:**
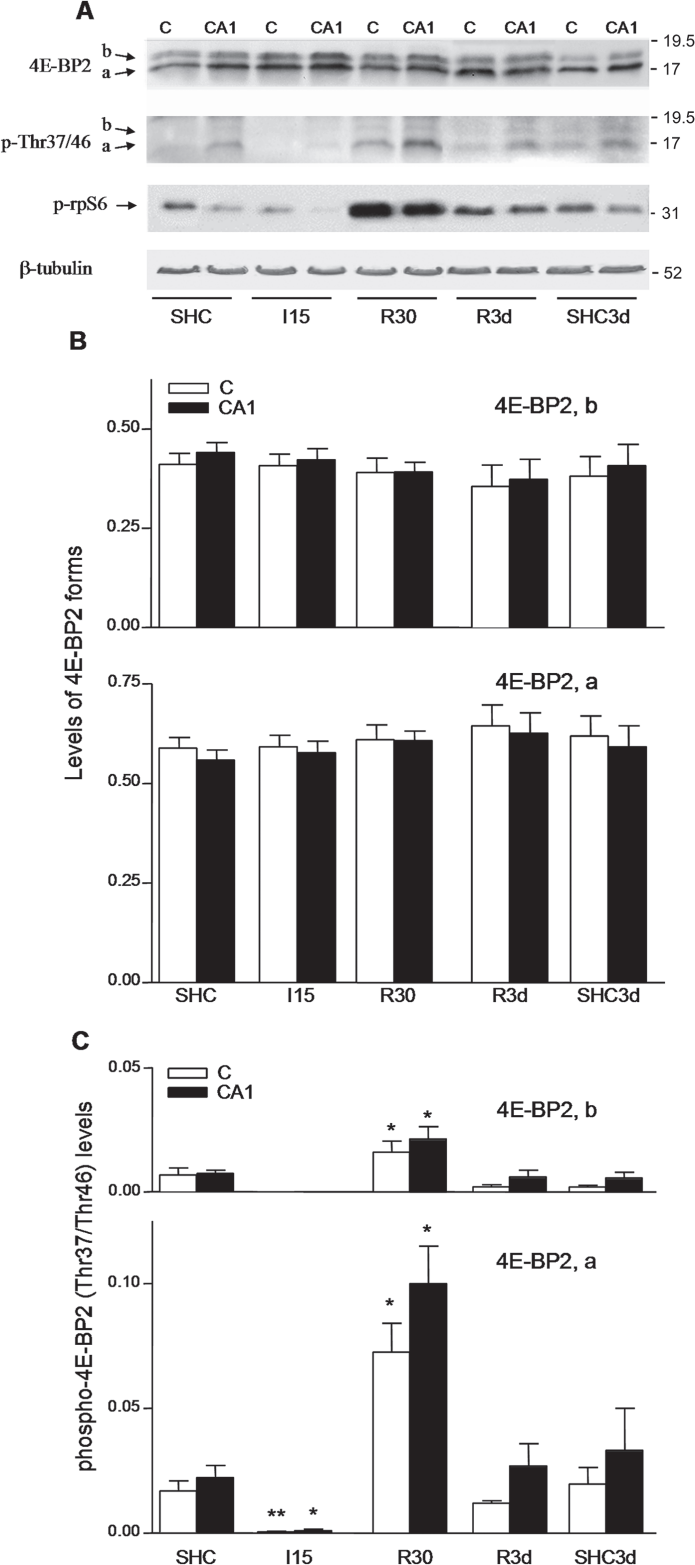
Identification of 4E-BP2 forms and their phosphorylation status in ischemia and IR stress. (A) Samples of the cerebral cortex, C, or hippocampal CA1 region, CA1, from control (SHC and SHC3d) and ischemic animals, without (I15) or with reperfusion (R30 and R3d), were subjected to western blotting with anti-4E-BP2 (4E-BP2), anti-phospho-4E-BP2 Thr^37^/Thr^46^ (p-Thr37/46) and anti-phospho-rpS6 Ser^235/236^ (p-rpS6) antibodies. Arrows show the *a* and *b* forms of 4E-BP2 and phospho-rpS6. Figures show representative results. The right numbers represent the apparent MW in kDa from protein markers. (B) Levels of the *a* and *b* forms of 4E-BP2 under ischemia and IR. Data are the quantification of the *a* and *b* forms (upper and lower bar graphs, respectively) with respect to total 4E-BP2 levels (ratios) and represented in arbitrary units; error bars indicate SEM. p > 0.05, compared with the SHC and SHC3d controls. (C) Quantification of the 4E-BP2 phosphorylation at Thr^37^/Thr^46^ residues induced by ischemia and reperfusion stress. Data are the quantification of the *a* and *b* phospho-forms (upper and lower bar graphs, respectively) detected with anti-phospho-4E-BP2 antibody with respect to total 4E-BP2 levels (ratios) and represented in arbitrary units; error bars indicate SEM. *p < 0.05; **p < 0.01, compared with the controls. All data were from four to six different animals and run in duplicate, and analyzed by ANOVA and ad hoc post-test, unless otherwise stated.

We investigated the physiological regulation of 4E-BP2 by phosphorylation, using control, ischemic and IR stress conditions in brain tissue. In rat brain samples, we resolved 4E-BP2 by SDS-PAGE and western blotting as two bands, which were named as *a* and *b* forms in order of decreasing electrophoretic mobility (Fig. 1A). The quantification data showed a proportion of 3:2 for the *a* and *b* forms of 4E-BP2, respectively (Fig. 1B). Ischemia (I15) and the subsequent reperfusion of 30 min or 3 days (R30 and R3d, respectively; also termed as short- and long-term reperfusion, respectively) did not induce any significant effect on the levels of 4E-BP2 forms and there were no changes between the cerebral cortex and hippocampal CA1 region (Fig. 1A, 4E-BP2; Fig. 1B). The 4E-BP2 phosphorylation state was investigated using a specific antibody for phospho-Thr^37^/phospho-Thr^46^ of 4E-BP1 that specifically recognizes these sites in 4E-BP2 [19]. The Thr^46^ region of 4E-BP2 is completely conserved to that of 4E-BP1, and the region around Thr^37^ between 4E-BP1 and 4E-BP2, although are not completely conserved, show a high sequence similarity (see S4 Fig.). Detection of phospho-Thr^37^/Thr^46^ in 4E-BP2 in western blot analysis was absent in the presence of phospho-Thr^37^/Thr^46^ blocking peptide (S1A Fig.). Fortunately, phospho-4E-BP1 and -4E-BP2 could be distinguished by their different electrophoretic mobility under the electrophoresis conditions used in this work (S1B Fig.). Phosphorylation at Thr^37^/Thr^46^ was detected in the *a* form of 4E-BP2 and residually in the *b* form (Fig. 1A, p-Thr37/46). I15 induced significant dephosphorylation at Thr^37^/Thr^46^, while R30 induced significant phosphorylation at Thr^37^/Thr^46^, predominantly in the *a* form, when compared with the control (Fig. 1A, p-Thr37/46; Fig. 1C). This phosphorylation did not induce any change in their electrophoretic mobility or in the levels of the *a* and *b* forms. R3d did not have any significant effect on 4E-BP2 phosphorylation with respect to the control and no significant changes were seen between the cerebral cortex and CA1 region, although the CA1 region showed higher 4E-BP2 phosphorylation at the analyzed sites under different experimental conditions (Fig. 1A, p-Thr37/46; Fig. 1C).

We studied the phosphorylation induced by the PI3K-Akt-mTOR signaling pathway on the ribosomal protein S6 (rpS6), a downstream target sensitive to mTOR activity [19]. The phosphorylation of rpS6 at Ser^235/236^ was significantly induced upon reperfusion in R30 (Fig. 1A, p-rpS6; see also S2 Fig.); whereas rpS6 phosphorylation decreased with long-term reperfusion (R3d) without significant changes between the cerebral cortex and CA1 (Fig. 1A, p-rpS6; S2 Fig.).

### 4E-BP2 analysis by 2-DGE

**Fig 2:**
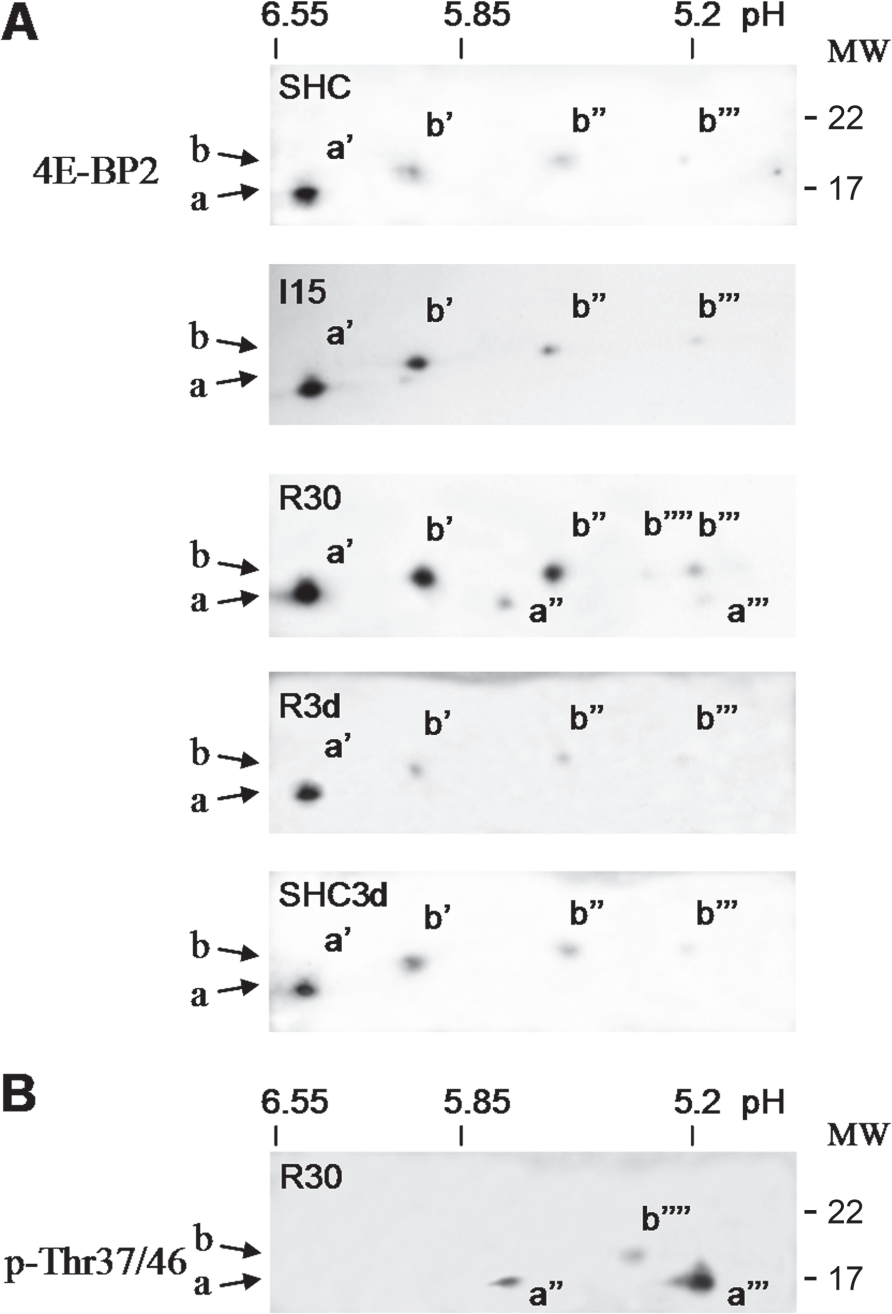
Analysis of 4E-BP2 by two-dimensional gel electrophoresis and changes induced by ischemia and IR stress. (A) Samples of cerebral cortex from control (SHC and SHC3d) and ischemic animals, without (I15) or with reperfusion (R30 and R3d), were subjected to two-dimensional gel electrophoresis (2-DGE) and western blotting with an anti-4E-BP2 antibody. The antibody-reactive spots were *a*’, *a*”, *a*”’, *b*’, *b*”, *b*”’ and *b*””. Figures show representative results from six different animals. (B) Identification of the phosphorylation sites for 4E-BP2 spots. R30 sample as in A was subjected to 2-DGE and western blotting for phospho-specific antibodies against 4E-BP2 Thr^37^/Thr^46^ (p-Thr37/46). The figure shows a representative result from three different animals. Arrows indicate the relative position of the *a* and *b* forms of 4E-BP2 in the molecular weight (MW) axis. MW is indicated in kDa.

To characterize further the physiological 4E-BP2 (phospho)forms induced by ischemia and upon reperfusion, we analyzed 4E-BP2 by two-dimensional gel electrophoresis and western blotting. The study was performed in cortical samples as no significant differences were found between the cerebral cortex and the CA1 region (see above). In the first dimension, pH 3–10 strips with a nonlinear gradient were used which improved the resolution in the pH 5–6.5 region that corresponded to the theoretical pI range of 4E-BP2. Four different spots were detected in all samples: a most basic spot (pI = 6.4), *a*’, that corresponded to the *a* form of 4E-BP2; and three spots, *b*’, *b*” and *b*”’ (pI = 6.05, 5.55 and 5.2) that corresponded to the *b* form (Fig. 2A). The pI of the spot *a*’ was close to the theoretical pI (6.5) of 4E-BP2 (computed in http://www.bioinformatics.org/sms2/). R30 induced two additional spots detected in the *a* form, *a*” and *a*”’ (pI = 5.67 and 5.15), and one additional spot between *b*” and *b*”’, *b*”” (pI = 5.3) (Fig. 2A, R30). No differences were found in the identified spots in the I15, R3d and control groups (Fig. 2A). Phosphorylation at the Thr^37^/Thr^46^ sites was only detected in R30, mainly in the *a*”’ spot, although there was also a reaction in both the *a*” and *b*”” spots, but not in the *a*’, *b*’, *b*” and *b*”’ spots (Fig. 2B). These results showed that the *a* form corresponded to one basic *a*’ spot dephosphorylated at Thr^37^/Thr^46^, and two more acidic spots, *a*” and *a*”’ with phospho-Thr^37^/Thr^46^ detected in the R30 group. The *b* form included three spots, *b*’, *b*” and *b*”’ that were dephosphorylated at Thr^37^/Thr^46^, and a residual *b*”” spot with a phosphorylation at Thr^37^/Thr^46^ detected in the R30 group (Figs. 1A and 2). Specific monoclonal anti-phospho-4E-BP1 (Ser^65^ 174A9) and rabbit polyclonal anti-phospho-4E-BP1 (Thr^70^) antibodies (Cell Signaling) were probed and no signal was detected on 4E-BP2 (M.I. Ayuso and A. Alcazar, unpublished observations).

### Association of 4E-BP2 to eIF4E

**Fig 3:**
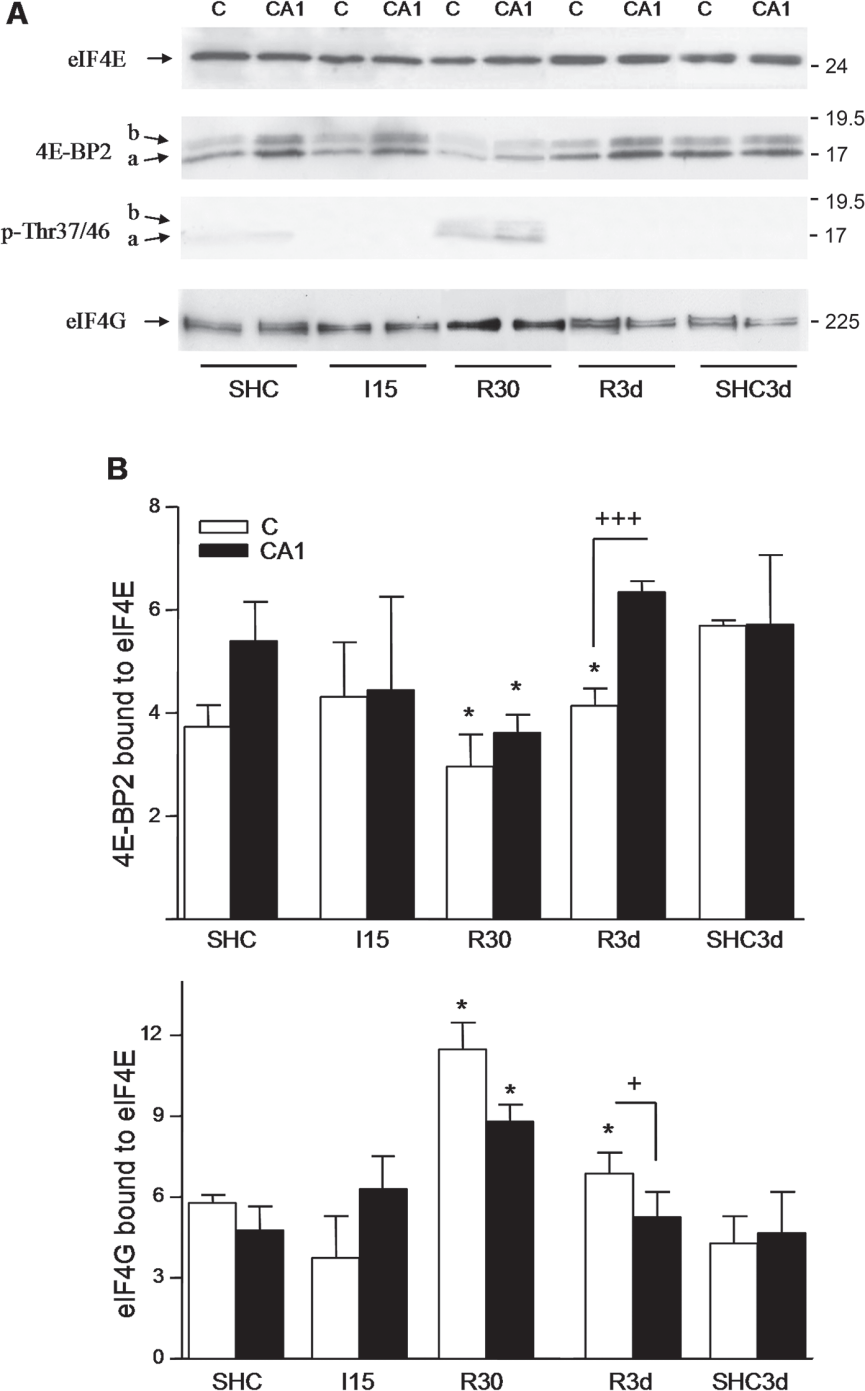
4E-BP2 and eIF4G association with eIF4E. (A) Samples as in Fig. 1A, were bound to m^7^GTP-Sepharose and analyzed by western blotting with anti-eIF4E (eIF4E), anti-4E-BP2 (4E-BP2), anti-phospho-4E-BP2 Thr^37^/Thr^46^ (p-Thr37/46) and anti-eIF4G (eIF4G) antibodies. Arrows show the relative position of eIF4E, the *a* and *b* forms of 4E-BP2, and eIF4G. The right numbers represent the apparent MW in kDa from protein markers. (B) Quantification of 4E-BP2 and eIF4G bound to eIF4E induced by ischemia and IR stress. No significant differences in the eIF4E levels were found (p ≥ 0.360). Data are the quantification of bound 4E-BP2 (*a* + *b* forms; upper bar graph), or bound eIF4G (lower bar graph), with respect to eIF4E levels (ratios) detected with the corresponding antibody and represented in arbitrary units. Error bars indicate SEM. *p < 0.05, compared with the controls; ^+^ p < 0.05, ^+++^p < 0.001, cerebral cortex, C, compared with the hippocampal CA1 region, CA1. Differences in eIF4G between R3d and SHC3d groups were done by two-tailed t test of data from six to eight different animals run in duplicate.

**Fig 4:**
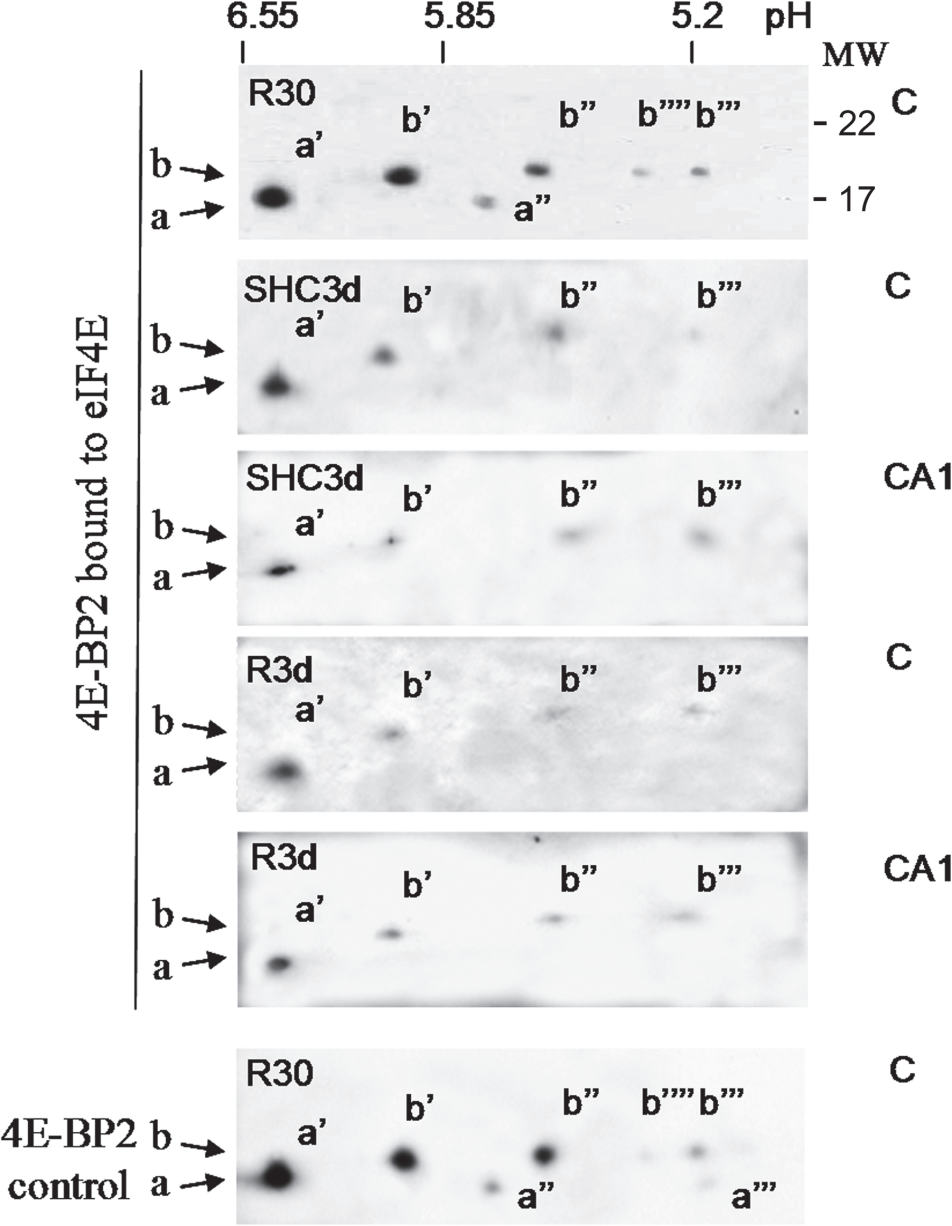
Analysis of 4E-BP2 associated to eIF4E by (2-DGE). Samples from m^7^GTP-Sepharose as in Fig. 3 were subjected to 2-DGE and western blotting and 4E-BP2 spots bound to eIF4E detected with an anti-4E-BP2 antibody (4E-BP2). Figures show the representative results from three different animals. Spots detected in whole samples (Fig. 2A) are shown as control (4E-BP2 control). MW is indicated in kDa.

The eIF4E is identified by its ability to bind to the 5'-cap structure and was consequently isolated by affinity chromatography in m^7^GTP-Sepharose, a cap-containing matrix [15]. Using this approach, we studied the 4E-BP2 bound to eIF4E, and the binding of eIF4G to eIF4E (eIF4F complex formation). Our results showed that both 4E-BP2 *a* and *b* were bound to eIF4E (Fig. 3A, 4E-BP2). R30 induced a significant decrease in 4E-BP2 bound to eIF4E when compared with controls, and accordingly, induced a significant increase in the binding of eIF4G to eIF4E (Fig. 3B). 4E-BP2 phosphorylation at Thr^37^/Thr^46^ was detected in the 4E-BP2 bound to eIF4E in R30 (Fig. 3A, p-Thr37/46). The analysis by 2-DGE of 4E-BP2 bound to eIF4E in R30 detected the *a*’, *a*” and all *b* spots, but not the *a*”’ spot (Fig. 4, R30). Note that the not detected *a*”’ spot corresponded to the most phosphorylated 4E-BP2 form in R30 (Fig. 2B), a condition where 4E-BP2 phosphorylation had been induced (Fig. 1). The phosphorylation at Thr^37^/Thr^46^ in the *a*” and *b*”” spots was assessed with anti-phospho-Thr^37^/Thr^46^ antibody (not shown). Therefore, in R30, the binding of 4E-BP2 to eIF4E decreased in parallel with the phosphorylation of 4E-BP2, although a fraction of the phosphorylated 4E-BP2 was found bound to eIF4E.

### Phosphorylation-independent dissociation of 4E-BP2 from eIF4E

**Fig 5:**
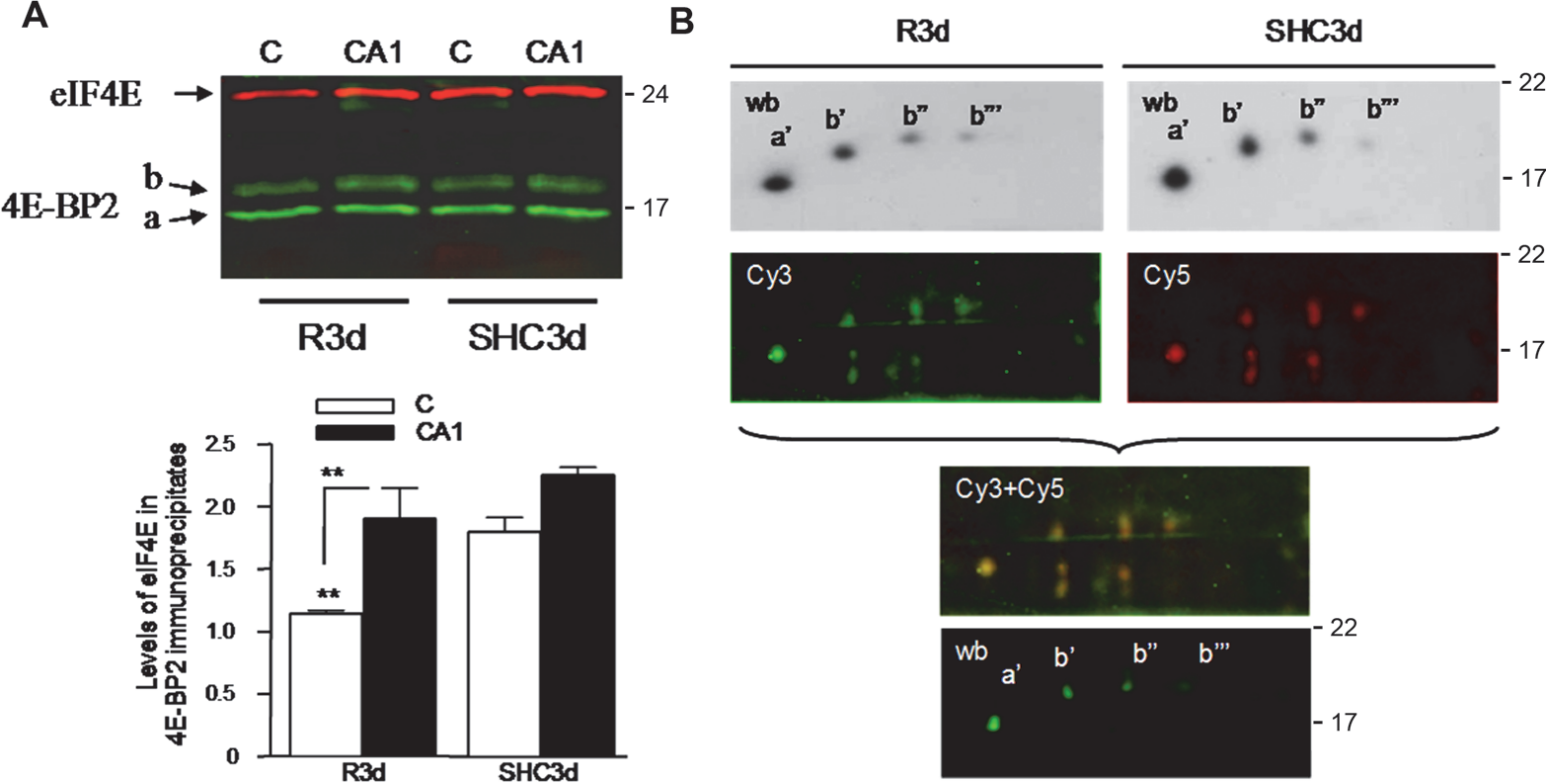
Analysis of 4E-BP2 immunoprecipitates. (A) Samples of cerebral cortex, C, or CA1 from control (SHC3d) and ischemic animals with long-term reperfusion (R3d), were immunoprecipitated with anti-4E-BP2 antibody and analyzed by western blotting with: anti-eIF4E antibody and anti-mouse IRDye 680LT-conjugated as secondary antibody (in red); and anti-4E-BP2 antibody and anti-rabbit IRDye 800CW-conjugated (in green). Bar graph shows the quantification of eIF4E in the immunoprecipitates; no significant differences in the 4E-BP2 levels were found (p ≥ 0.520). (B) 4E-BP2 immunoprecipitates of cerebral cortex from R3d and SHC3d control, were subjected to 2-DGE and western blotting (wb) with anti-4E-BP2 antibody (in grey); or were labeled with Cy3- and Cy5-dye, subjected to 2-DGE and after analyzed with an fluorescence imager (Cy images) and by western blotting with anti-4E-BP2 antibody as in (A) (wb, in green). The right numbers represent the apparent MW in kDa from protein markers.

In addition to R30, long-term reperfusion (R3d) induced a significant decrease in the binding of 4E-BP2 to eIF4E in the cerebral cortex compared with SHC3d control (Fig. 3). Decrease that was very significant in comparison with the binding in the CA1 region and accompanied by an increase in eIF4G bound to eIF4E in the cerebral cortex compared with the control or CA1 (Fig. 3). This result was confirmed in 4E-BP2 immunoprecipitates, wherein R3d showed a significant decrease in the eIF4E associated to 4E-BP2 in the cerebral cortex compared with SHC3d control or CA1 (Fig. 5A). However, no changes were found in 4E-BP2 phosphorylation (Figs. 1 and 3A, p-Thr37/46). To study if other possible 4E-BP2 forms may modulate the binding to eIF4E in R3d, we analyzed by 2-DGE the 4E-BP2 bound to eIF4E in m^7^GTP-Sepharose. The results showed that identical *a*’, *b*’, *b*” and *b*”’ forms of 4E-BP2 were bound to eIF4E in the SHC3d control or R3d in both cerebral cortex and CA1 region, with no differences between them (Fig. 4). These results were confirmed analyzing the immunoprecipitated 4E-BP2 by 2-DGE (Fig. 5B). 4E-BP2 immunoprecipitates were labelled with Cy-dyes, resolved in 2-DGE, and 4E-BP2 identified and visualized with a confocal fluorescence imager (Fig. 5B). All these experiments detected identical *a*’, *b*’, *b*” and *b*”’ forms of 4E-BP2 in the cerebral cortex in R3d and SHC3d control without differences between them; forms that corresponded to unphosphorylated 4E-BP2 (Fig. 2) and able to bind to eIF4E (Fig. 4). In summary, upon long-term reperfusion, there was a decrease in the binding of 4E-BP2 to eIF4E in the cerebral cortex, effects that occurred without changes in 4E-BP2 phosphorylation.

**Fig 6:**
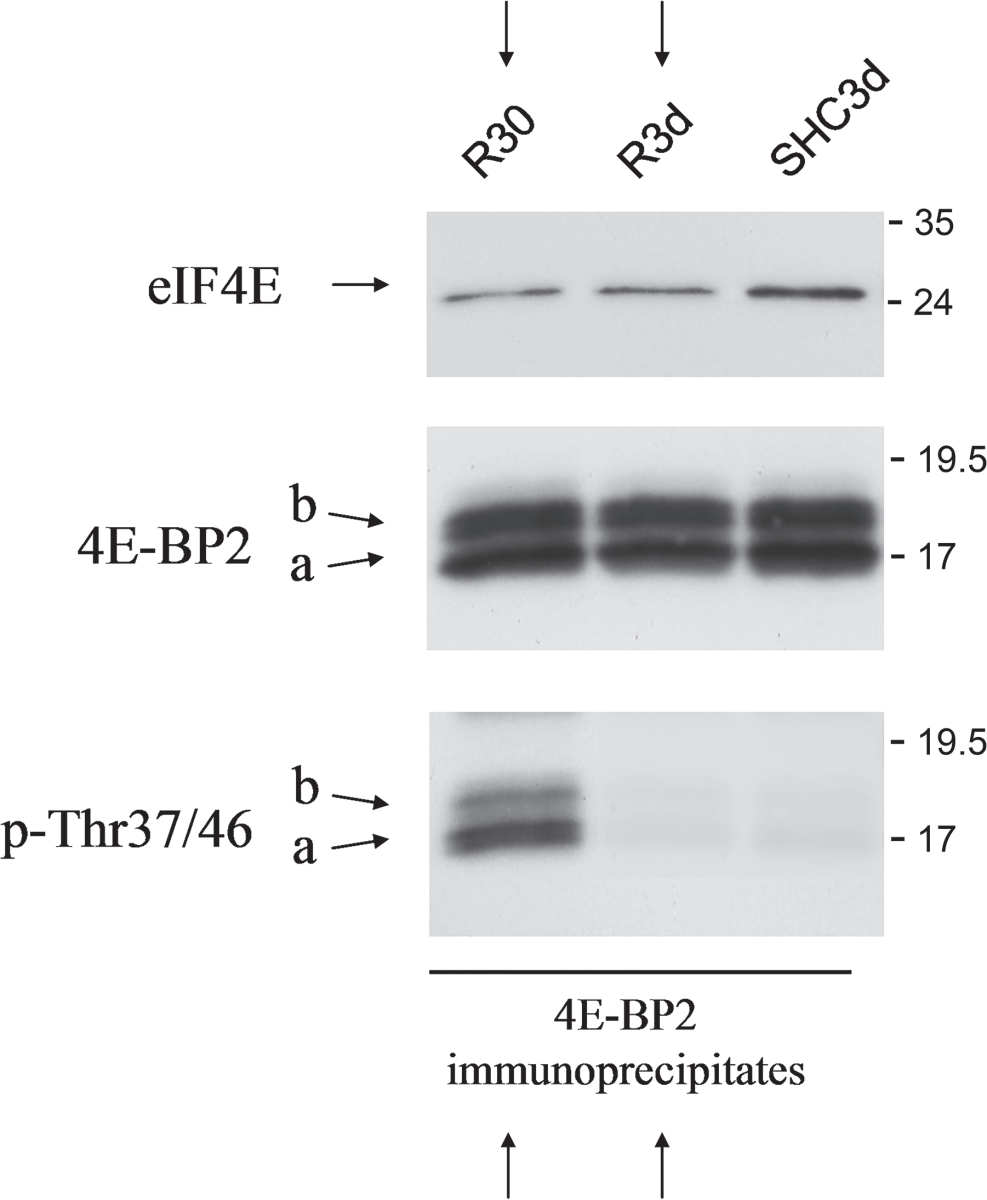
Changes in 4E-BP2/eIF4E association dependent or independent of 4E-BP2 phosphorylation. Samples of cerebral cortex from control (SHC3d) and ischemic animals with short- and long-term reperfusion (R30 and R3d, respectively), were immunoprecipitated with anti-4E-BP2 antibody and analyzed by western blotting with anti-eIF4E (eIF4E), anti-4E-BP2 (4E-BP2), and anti-phospho-4E-BP1/2 Thr^37^/Thr^46^ (p-Thr37/46) antibodies. The right numbers represent the apparent MW in kDa from protein markers. The figure shows similar decreased levels of associated eIF4E compared with the control (upper arrows). However, 4E-BP2 was phosphorylated at Thr^37^/Thr^46^ in R30, and not in R3d (lower arrows).

To highlight the specific changes in 4E-BP2/eIF4E association dependent and independent of 4E-BP2 phosphorylation demonstrated here, we accomplished the Fig. 6. The figure shows 4E-BP2 immunoprecipitates from R30 and R3d with similar decreased levels of the associated eIF4E with respect to control. However, 4E-BP2 was phosphorylated at Thr^37^/Thr^46^ in R30, and not in R3d (Fig. 6).

## Discussion

In order to know the regulation of 4E-BP2 in the brain, we report a physiological study of 4E-BP2 analyzing the levels and phosphorylation status in control conditions and a stress-induced translational repression condition, IR stress. Two 4E-BP2 forms were resolved in rat brain under SDS-PAGE and named *a* and *b* in order of decreasing electrophoretic mobility. Other authors have described three forms of 4E-BP2 by SDS-PAGE in the mouse brain, identifying the two forms with slower mobility—a double band—as deamidated forms of 4E-BP2 at Asn^99^/Asn^102^ that were resolved in three spots by 2-DGE [19]. These spots correspond with the *b’*, *b”* and *b”’* spots described here, and therefore, we concluded that the *b* form corresponds with the deamidated forms of 4E-BP2 and that they can be resolved by SDS-PAGE into single or double bands, depending of electrophoretic conditions.

Because 4E-BP1 in brain is resolved in three bands with decreasing electrophoretic mobility that correspond with increasing phosphorylation status [8, 16], the first hypothesis was that the *b* form would be (hyper)phosphorylated 4E-BP2 and the *a* form would be un- or hypo-phosphorylated 4E-BP2. To confirm this, we studied the phosphorylation at Thr^37^/Thr^46^ sites. Unlike 4E-BP1, 4E-BP2 phosphorylation on Thr^37^/Thr^46^ was poorly detected in the *b* form, and was mainly detected at the higher mobility form in two spots, *a”* and *a”’*, without changes in the apparent molecular weight of 4E-BP2. So far, the results showed two forms of 4E-BP2, one with a lower electrophoretic mobility and another with higher electrophoretic mobility that was susceptible to phosphorylation at Thr^37^/Thr^46^ sites. Phosphorylation that was resolved into more acidic spots by 2-DGE. Ischemia and subsequent reperfusion induced dephosphorylation and phosphorylation of 4E-BP2, respectively. This phosphorylation was restored to control levels upon long-term reperfusion.

IR stress during short-term reperfusion (R30) induced phosphorylation of 4E-BP2 at Thr^37^/Thr^46^, in parallel with a decrease in the 4E-BP2 bound to eIF4E and a significant increase in the binding of eIF4G to eIF4E. Further, the more acidic form of phosphorylated 4E-BP2 did not bind to eIF4E. The induced 4E-BP2 phosphorylation was correlated with the increased phosphorylation of rpS6 at Ser^235/236^, a sign of mTORC1 activity. These results agree with the mTORC1-dependent 4E-BP1 phosphorylation at Thr^37^/Thr^46^ [6], with the previously described restoration of mTOR activity and phosphorylation of 4E-BP1 in brain upon ischemic reperfusion [8], and with the established mechanism to 4E-BPs, where the phosphorylation of 4E-BPs reduces their affinity for eIF4E and allows eIF4E to bind eIF4G [5–8].

In summary, the experiments demonstrated that: (i) 4E-BP2 was resolved in SDS-PAGE into forms that are apparently independent of phosphorylation; (ii) the highest migrating form was the principal 4E-BP2 form susceptible to phosphorylation at Thr^37^/Thr^46^ sites; (iii) this phosphorylation did not induce any shifts in their apparent molecular weight; (iv) all detected unphosphorylated forms of 4E-BP2 bound to eIF4E; and (v) 4E-BP2 phosphorylation at Thr^37^/Thr^46^ allowed the binding to eIF4E. The last result was confirmed in primary neuronal cultures subjected to ischemia *in vitro*, where phosphorylation at Thr^37^/Thr^46^ was detected in the 4E-BP2 bound to eIF4E (S3 Fig.). However, (hyper)phosphorylated 4E-BP2—the *a”’* form—detected in R30 does not bind to eIF4E, suggesting that phosphorylation of 4E-BP2 would be sequential for prevention the binding to eIF4E. As similarly described for 4E-BP1 [6], including brain tissue [8].

Interestingly, long-term reperfusion (R3d) induced a decrease in the 4E-BP2 bound to eIF4E in the cerebral cortex without increase in their phosphorylation levels or changes in the 4E-BP2 forms, as demonstrated the 2-DGE experiments. The dissociation of 4E-BP2 and eIF4E during long-term reperfusion was also demonstrated by affinity to the cap-containing matrix (this paper), in 4E-BP2 immunoprecipitates (this paper), and was in agreement with immunohistochemical studies in brain sections and in parallel to the increase of translational rates [13]. Besides, ischemia (I15) induced 4E-BP2 dephosphorylation and no changes were induced in the binding of 4E-BP2 to eIF4E with respect to the controls, nor in the binding between eIF4G and eIF4E. The absence of changes in 4E-BP2 phosphorylation in R3d was coincident with the observed in rpS6 phosphorylation, and in agreement with the previously described control values of both mTOR activity and 4E-BP1 phosphorylation in brain at long-term reperfusion [8]. All these results suggest that there is another mechanism distinct from 4E-BP2 phosphorylation that interferes with the binding of 4E-BP2 to eIF4E under long-term ischemic reperfusion stress in brain. This hypothesis is also supported by Cy-labeled 2-DGE experiments, where other potential post-translational modifications—e.g., 4E-BP2-phosphorylation at Ser^65^ and Thr^70^—were not detected on 4E-BP2 (MIA and AA, unpublished observations) during long-term reperfusion in the cerebral cortex compared with the control condition or CA1.

We study if eIF4E –the other partner in 4E-BP2/eIF4E association—was implicated in ischemia and IR stress, or correlated with 4E-BP2/eIF4E association. We analyzed the phosphorylation levels at the regulated Ser^209^ site [7] with a rabbit polyclonal anti-phospho-eIF4E (Ser^209^) antibody (Cell Signaling #9741). No significant changes in eIF4E phosphorylation were found in R3d between the cerebral cortex and CA1 at R3d or when compared with the control (not shown). We also consider eIF4GI and II as potential interaction factors. We studied eIF4GI and II levels, and we did not find differences between the cerebral cortex and CA1 or controls (not shown). In addition, we studied the phosphorylation of eIF4G, but only anti-phospho-Ser1108, anti-phospho-Ser1186, and anti-phospho-Ser1232 antibodies are available against rat, whereas several and multiple phosphorylation sites are described in human eIF4GI and II [20, 21]. To date, our results are inconclusive.

The results present here show the (hyper)phosphorylation of 4E-BP2 and the inhibition of the binding to eIF4E upon reoxygenation after ischemia (R30), when mTOR signaling is activated [7]. Interestingly, at long-term reperfusion there is a release of 4E-BP2 from eIF4E independent of 4E-BP2 phosphorylation in the cerebral cortex. Result that was in parallel with an increase in the binding of eIF4G to eIF4E. However, the eIF4G bound to eIF4E was lower than in R30. We hypothesize that a potential modulator could compete with 4E-BP2 and release eIF4E, modulator that could also interfere in the binding of eIF4G to eIF4E (e.g., through an eIF4E-analogue binding domain). These findings of changes in 4E-BP2/eIF4E association dependent and independent of 4E-BP2 phosphorylation were illustrated in the Fig. 6. Therefore, a phosphorylation-dependent and-independent regulation of 4E-BP2 can be feasible. The reoxygenation after ischemia and the long-term reperfusion are conditions not comparable. The reoxygenation is a stressed condition where the cells needs trigger mechanism to cell survival such as protein synthesis induction, and mTOR activity restoration would play the critical signaling to cell recovery. In the long-term reperfusion, when the delayed neuronal death occurs in the selectively vulnerable neurons (e.g., the hippocampal CA1 neurons), there is a persistent protein synthesis inhibition in these neurons [10, 11, 22], situation that does not occur in viable neurons by an unknown translational control. Thus, neuronal cells from cerebral cortex response to IR stress with the dissociation of 4E-BP2 and eIF4E, and they are resistant to ischemic reperfusion. Finally, these results demonstrate compelling evidence that the paradigm of 4E-BPs regulation by phosphorylation may not be the unique for 4E-BP2, and we propose that other potential modulators might regulate the binding of 4E-BP2 to eIF4E. Further studies are necessary to elucidate a regulation of 4E-BP2 independent of phosphorylation.

## Supporting Information

S1 Fig Phospho-4E-BP1 and -4E-BP2 are detected by anti-phospho-Thr37/46 antibodies in rat brain with different electrophoretic mobility. Control and ischemic-reperfusion brain samples were subjected to SDS-PAGE and western blot. (A) The membrane was probed with anti-phospho-4E-BP1/2 Thr^37^ and/or Thr^46^ antibody (p-Thr37/46; upper panel) in the absence (left panel) or presence (right panel) of phospho-4E-BP1 (Thr^37^/Thr^46^) blocking peptide (#1052 from Cell Signaling), and re-probed with anti-4E-BP2 (lower panel) antibody. (B) The membrane was probed with anti-phospho-4E-BP1/2 Thr^37^ and/or Thr^46^ (p-Thr37/46; upper panel), and re-probed with anti-4E-BP1 (middle panel) and anti-4E-BP2 (lower panel) antibodies to exact identification. The results show that β and γ forms of 4E-BP1 and “a” and “b” forms of 4E-BP2 are susceptible to detection by the anti-phospho-Thr^37^/Thr^46^ antibody in rat brain. Under the electrophoretic conditions of this work (see Experimental section), “beta” and “gamma” forms of 4E-BP1 are resolved at 20 and 21 kDa, respectively, whereas “a” and “b” forms of 4E-BP2 are resolved at 17 and 18.5 kDa, respectively. These differences are sufficient to distinguish one from another. The right numbers indicate the apparent molecular weight (MW) in kDa from protein markers.
(TIF)

S2 Fig Quantification of ribosomal protein S6 (rpS6) phosphorylation at Ser^235/236^ induced by ischemia and reperfusion stress. Samples of the cerebral cortex, C, or hippocampal CA1 region, CA1, from control (SHC and SHC3d) and ischemic animals, without (I15) or with reperfusion (R30 and R3d), were subjected to western blotting with anti-phospho-rpS6 Ser^235/236^ (p-rpS6) and anti-rpS6 (rpS6) antibodies. Arrows indicate the detected phospho-rpS6 and rpS6. The right numbers indicate the apparent MW in kDa from protein markers. No significant differences in the rpS6 levels were found (p ≥ 0.234, by ANOVA test for all comparisons between experimental groups). Data (bar graph) are the quantification of phospho-rpS6 with respect to total rpS6 levels (ratios) from four to six different animals run in duplicate and represented in arbitrary units; error bars indicate SEM. *p < 0.05, compared with the controls.
(TIF)

S3 Fig Identification of the 4E-BP1/2 phosphorylation sites in neuronal cells. Primary neuronal cells in culture (control) [23] were subjected to oxygen‒glucose deprivation for 4 h to induce ischemia (I4h) and then maintained in control culture condition for 24 h to recovery (R24h). The cells were then lysed as described in the cited reference. (A) Cell lysates were subjected to western blotting for anti-eIF4E (eIF4E), anti-4E-BP2 (4E-BP2), and anti-phospho-4E-BP1/2 Thr^37^/Thr^46^ (p-Thr37/46) antibodies. (B) Alternatively, cell lysates were bound to m^7^GTP-Sepharose and analyzed by western blotting as above described. Arrows show the β and γ positions for 4E-BP1, and the *a* and *b* forms of 4E-BP2. Note that phosphorylation at Thr^37^/Thr^46^ was detected in the 4E-BP2 bound to eIF4E in the cap-containing matrix (m^7^GTP-Sepharose), but this phosphorylation was not present for 4E-BP1, as it was described previously [8]. The right numbers indicate the apparent MW in kDa from protein markers.
(TIF)

S4 Fig Alignment of the sequences of 4E-BP1 and 4E-BP2 in human and rat. Identical amino acids between 4E-BP1 and 4E-BP2 are marked in blue and yellow in the human and rat sequence, respectively. Homology between human and rat is marked in bold type. The phosphorylation regulation sites are marked in red; the amino acids susceptible to deamidation in green. The eIF4E binding site [24] is boxed in black; the TOS motif [25] in blue; and the RAIP sequence [26] in red. Sequences were obtained from UniProtKB database (http://www.uniprot.org/).
(TIF)

## References

[1] White BC, Sullivan JM, DeGracia DJ, O'Neil BJ, Neumar RW, Grossman LI, et al Brain ischemia and reperfusion: molecular mechanisms of neuronal injury. J Neurol Sci. 2000;179: 1–33. 11054482 10.1016/s0022-510x(00)00386-5

[2] Warner DS, Sheng H, Batinic-Haberle I. Oxidants, antioxidants and the ischemic brain. J Exp Biol. 2004;207: 3221–3231. 15299043 10.1242/jeb.01022

[3] Lipton P. Ischemic cell death in brain neurons. Physiol Rev. 1999;79: 1431–1568. 10508238 10.1152/physrev.1999.79.4.1431

[4] Hershey JWB, Merrick WC. The pathway and mechanism of initiation of protein synthesis In: Sonenberg N, Hershey JWB, Mathews MB, editors. Translational control of gene expression. New York: Cold Spring Harbor Laboratory Press 2000 pp. 33–88.

[5] Rhoads RE. eIF4E: New family members, new binding partners, new roles. J Biol Chem. 2009;284: 16711–16715. 10.1074/jbc.R900002200 19237539 PMC2719305

[6] Proud CG. Signalling to translation: how signal transduction pathways control the protein synthetic machinery. Biochem J. 2007;403: 217–234. 17376031 10.1042/BJ20070024

[7] Sonenberg N, Hinnebusch AG. Regulation of translation initiation in eukaryotes: mechanisms and biological targets. Cell. 2009;136: 731–745. 10.1016/j.cell.2009.01.042 19239892 PMC3610329

[8] Ayuso MI, Hernandez-Jimenez M, Martin ME, Salinas M, Alcazar A. New hierarchical phosphorylation pathway of the translational represor eIF4E-binding protein 1 (4E-BP1) in ischemia-reperfusion stress. J Biol Chem. 2010;285: 34355–34363. 10.1074/jbc.M110.135103 20736160 PMC2966049

[9] Tsukiyama-Kohara K, Vidal SM, Gingras AC, Glover TW, Hanash SM, Heng H, et al Tissue distribution, genomic structure, and chromosome mapping of mouse and human eukaryotic initiation factor 4E-binding proteins 1 and 2. Genomics. 1996;38: 353–363. 8975712 10.1006/geno.1996.0638

[10] Hossmann KA. Pathophysiology and therapy of experimental stroke. Cell Mol Neurobiol. 2006;26: 1057–1083. 16710759 10.1007/s10571-006-9008-1 PMC11520628

[11] Jamison JT, Kayali F, Rudolph J, Marshall M, Kimball SR, DeGracia DJ. Persistent redistribution of poly-adenylated mRNAs correlates with translation arrest and cell death following global brain ischemia and reperfusion. Neuroscience. 2008;154: 504–520. 10.1016/j.neuroscience.2008.03.057 18456413 PMC2494580

[12] Garcia-Bonilla L, Cid C, Alcazar A, Burda J, Ayuso I, Salinas M. Regulation proteins of eukaryotic initiation factor 2-alpha subunit (eIF2α) phosphatase, under ischemic reperfusion and tolerance. J Neurochem. 2007;103: 1368–1380. 17760864 10.1111/j.1471-4159.2007.04844.x

[13] Ayuso MI, Martinez-Alonso E, Cid C, de Leciñana MA, Alcazar A. The translational repressor eIF4E-binding protein 2 (4E-BP2) correlates with selective delayed neuronal death after ischemia. J Cereb Blood Flow Metabol. 2013;33: 1173–1181. 10.1038/jcbfm.2013.60 23591646 PMC3734765

[14] Martin de la Vega C, Burda J, Nemethova M, Quevedo C, Alcazar A, Martin ME, et al Possible mechanisms involved in the down-regulation of translation during transient global ischaemia in the rat brain. Biochem J. 2001;357: 819–826. 11463353 10.1042/0264-6021:3570819 PMC1222012

[15] Webb NR, Chari RV, DePillis G, Kozarich JW, Rhoads RE. Purification of the messenger RNA cap-binding protein using a new affinity medium. Biochemistry. 1984;23: 177–181. 6696877 10.1021/bi00297a001

[16] Quevedo C, Salinas M, Alcazar A. Regulation of Cap-dependent translation by insulin-like growth factor-1 in neuronal cells. Biochem Biophys Res Commun. 2002;291: 560–566. 11855825 10.1006/bbrc.2002.6479

[17] Cid C, Alvarez-Cermeño JC, Camafeita E, Salinas M, Alcazar A. Antibodies reactive to heat shock protein 90 induce oligodendrocyte precursor cell death in culture. Implications for demyelination in multiple sclerosis. FASEB J. 2004;18: 409–411. 14688203 10.1096/fj.03-0606fje

[18] Cid C, Garcia-Bonilla L, Camafeita E, Burda J, Salinas M, Alcazar A. Proteomic characterization of protein phosphatase 1 complexes in ischemia-reperfusion and ischemic tolerance. Proteomics. 2007;7: 3207–3218. 17683050 10.1002/pmic.200700214

[19] Bidinosti M, Ran I, Sanchez-Carbente MR, Martineau Y, Gingras AC, Gkogkas C, et al Postnatal deamidation of 4E-BP2 in brain enhances its association with raptor and alters kinetics of excitatory synaptic transmission. Mol Cell. 2010;37: 797–808. 10.1016/j.molcel.2010.02.022 20347422 PMC2861547

[20] Dobrikov M, Dobrikova E, Shveygert M, Gromeier M. Phosphorylation of eukaryotic translation initiation factor 4G1 (eIF4G1) by protein kinase Cα regulates eIF4G1 binding to Mnk1. Mol Cell Biol. 2011;31: 2947–2989. 10.1128/MCB.05589-11 21576361 PMC3133411

[21] Srivastava T, Fortin DA, Nygaard S, Kaech S, Sonenberg N, Edelman AM, et al Regulation of neuronal mRNA translation by CaM-kinase I phosphorylation of eIF4GII. J Neurosci. 2012;32: 5620–5630. 10.1523/JNEUROSCI.0030-12.2012 22514323 PMC3346851

[22] Hermann DM, Kilic E, Hata R, Hossmann KA, Mies G. Relationship between metabolic dysfunctions, gene responses and delayed cell death after mild focal cerebral ischemia in mice. Neuroscience. 2001;104: 947–955. 11457582 10.1016/s0306-4522(01)00125-7

[23] Quevedo C, Alcazar A, Salinas M. Two different signal transduction pathways are implicated in the regulation of initiation factor 2B activity in insulin-like growth factor-1-stimulated neuronal. J Biol Chem. 2000;275: 19192–19197. 10764740 10.1074/jbc.M000238200

[24] Lawrence JC Jr, Abraham RT. PHAS/4E-BPs as regulators of mRNA translation and cell proliferation. Trends Biochem Sci. 1997;22: 345–349. 9301335 10.1016/s0968-0004(97)01101-8

[25] Beugnet A, Wang X, Proud CG. Target of rapamycin (TOR)-signaling and RAIP motifs play distinct roles in the mammalian TOR-dependent phosphorylation of initiation factor 4E-binding protein 1. J Biol Chem. 2003;278: 40717–40722. 12912989 10.1074/jbc.M308573200

[26] Wang X, Beugnet A, Murakami M, Yamanaka S, Proud CG. Distinct signaling events downstream of mTOR cooperate to mediate the effects of amino acids and insulin on initiation factor 4E-binding proteins. Mol Cell Biol. 2005;25: 2558–2572. 15767663 10.1128/MCB.25.7.2558-2572.2005 PMC1061630

