# Supplementary figures and images for: Dissociation of eIF4E-Binding Protein 2 (4E-BP2) from eIF4E Independent of Thr37/Thr46 Phosphorylation in the Ischemic Stress Response
Source: PLoS One. 2015 Mar 30;10(3):e0121958. doi: 10.1371/journal.pone.0121958 (PMC4379021; doi:10.1371/journal.pone.0121958)

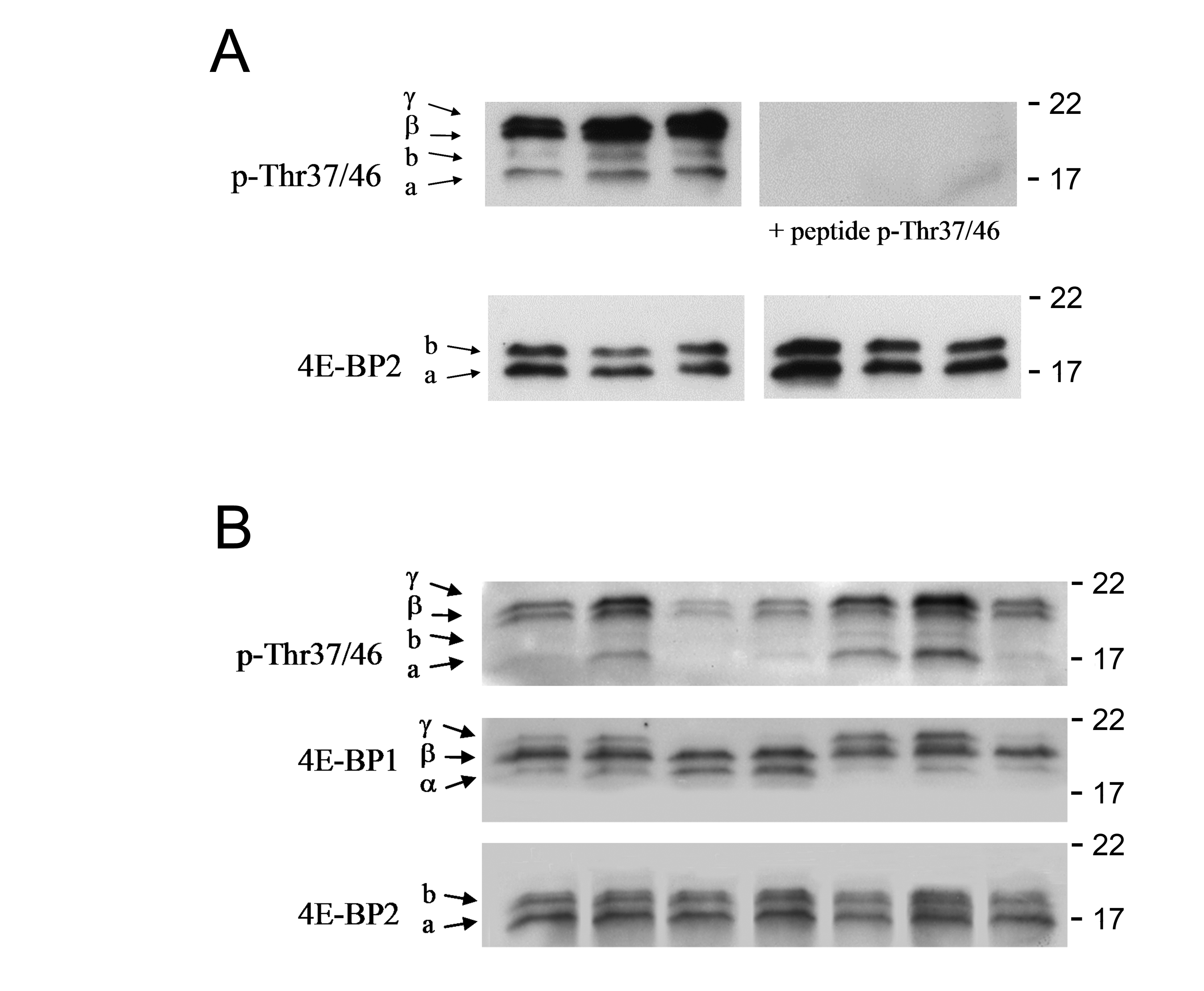

Supplement: S1 Fig — Control and ischemic-reperfusion brain samples were subjected to SDS-PAGE and western blot. (A) The membrane was probed with anti-phospho-4E-BP1/2 Thr37 and/or Thr46 antibody (p-Thr37/46; upper panel) in the absence (left panel) or presence (right panel) of phospho-4E-BP1 (Thr37/Thr46) blocking peptide (#1052 from Cell Signaling), and re-probed with anti-4E-BP2 (lower panel) antibody. (B) The membrane was probed with anti-phospho-4E-BP1/2 Thr37 and/or Thr46 (p-Thr37/46; upper panel), and re-probed with anti-4E-BP1 (middle panel) and anti-4E-BP2 (lower panel) antibodies to exact identification. The results show that β and γ forms of 4E-BP1 and “a” and “b” forms of 4E-BP2 are susceptible to detection by the anti-phospho-Thr37/Thr46 antibody in rat brain. Under the electrophoretic conditions of this work (see Experimental section), “beta” and “gamma” forms of 4E-BP1 are resolved at 20 and 21 kDa, respectively, whereas “a” and “b” forms of 4E-BP2 are resolved at 17 and 18.5 kDa, respectively. These differences are sufficient to distinguish one from another. The right numbers indicate the apparent molecular weight (MW) in kDa from protein markers. (TIF) [file pone.0121958.s001.tif]

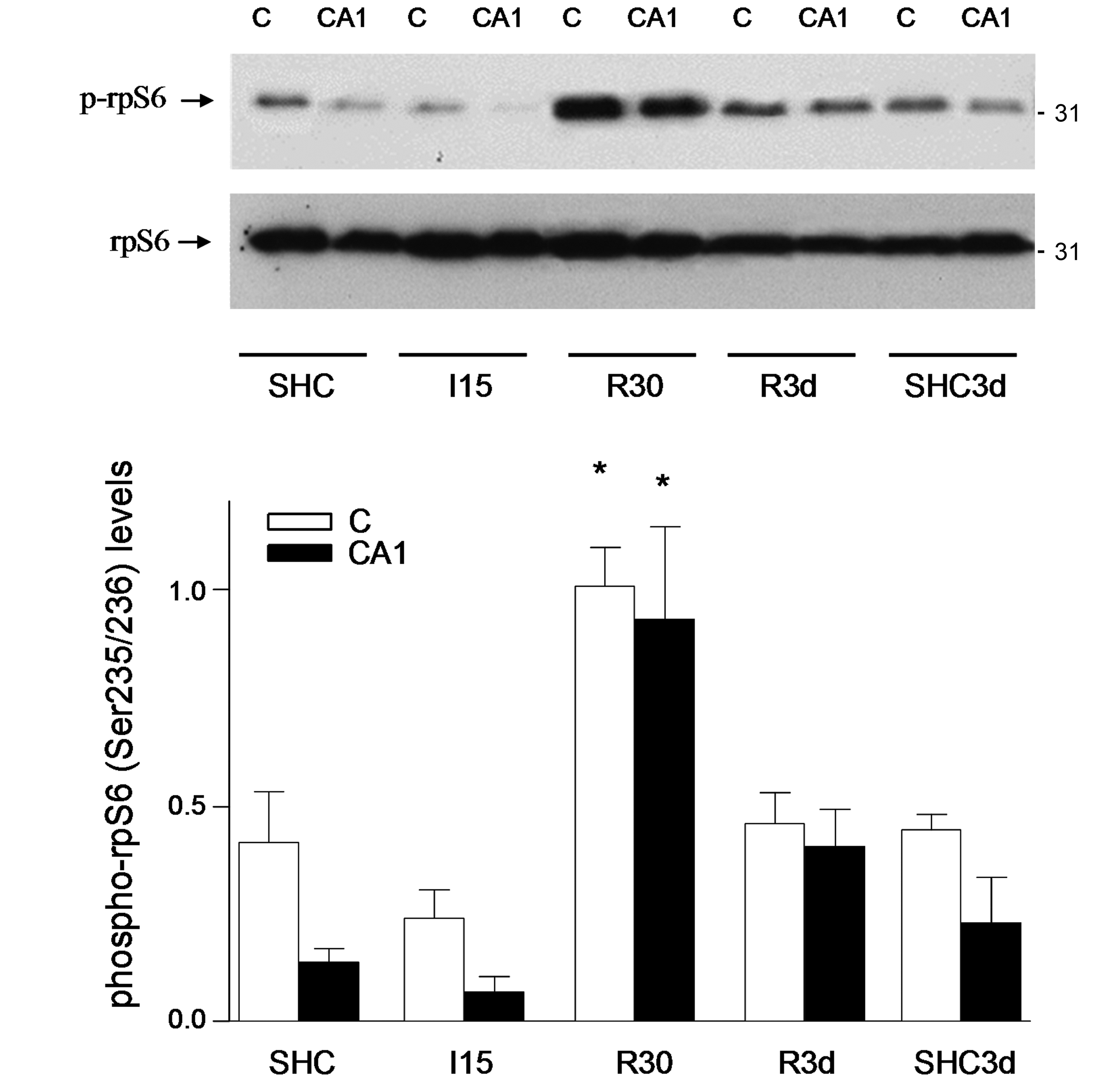

Supplement: S2 Fig — Samples of the cerebral cortex, C, or hippocampal CA1 region, CA1, from control (SHC and SHC3d) and ischemic animals, without (I15) or with reperfusion (R30 and R3d), were subjected to western blotting with anti-phospho-rpS6 Ser235/236 (p-rpS6) and anti-rpS6 (rpS6) antibodies. Arrows indicate the detected phospho-rpS6 and rpS6. The right numbers indicate the apparent MW in kDa from protein markers. No significant differences in the rpS6 levels were found (p ≥ 0.234, by ANOVA test for all comparisons between experimental groups). Data (bar graph) are the quantification of phospho-rpS6 with respect to total rpS6 levels (ratios) from four to six different animals run in duplicate and represented in arbitrary units; error bars indicate SEM. *p < 0.05, compared with the controls. (TIF) [file pone.0121958.s002.tif]

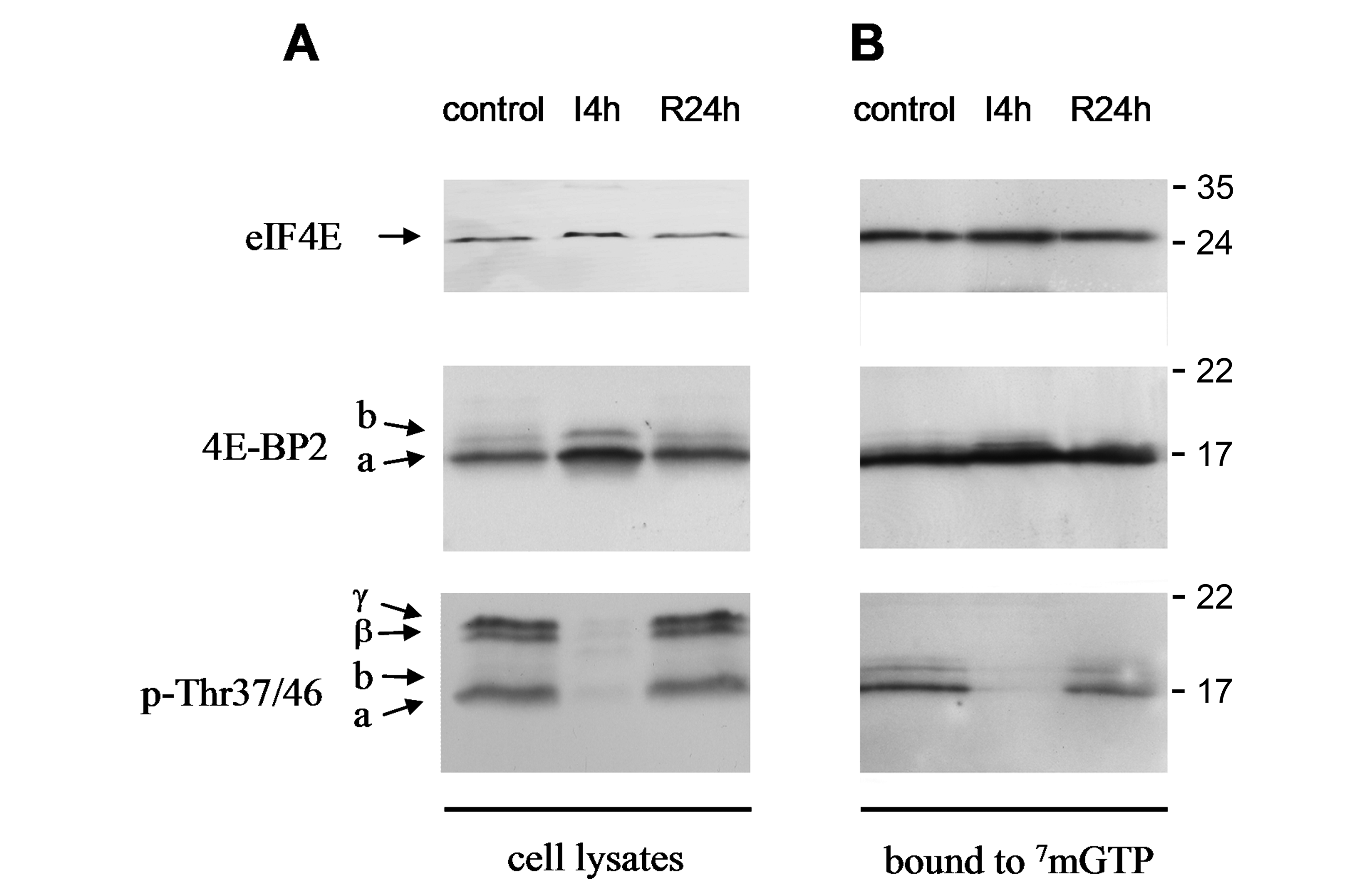

Supplement: S3 Fig — Primary neuronal cells in culture (control) [23] were subjected to oxygen‒glucose deprivation for 4 h to induce ischemia (I4h) and then maintained in control culture condition for 24 h to recovery (R24h). The cells were then lysed as described in the cited reference. (A) Cell lysates were subjected to western blotting for anti-eIF4E (eIF4E), anti-4E-BP2 (4E-BP2), and anti-phospho-4E-BP1/2 Thr37/Thr46 (p-Thr37/46) antibodies. (B) Alternatively, cell lysates were bound to m7GTP-Sepharose and analyzed by western blotting as above described. Arrows show the β and γ positions for 4E-BP1, and the a and b forms of 4E-BP2. Note that phosphorylation at Thr37/Thr46 was detected in the 4E-BP2 bound to eIF4E in the cap-containing matrix (m7GTP-Sepharose), but this phosphorylation was not present for 4E-BP1, as it was described previously [8]. The right numbers indicate the apparent MW in kDa from protein markers. (TIF) [file pone.0121958.s003.tif]

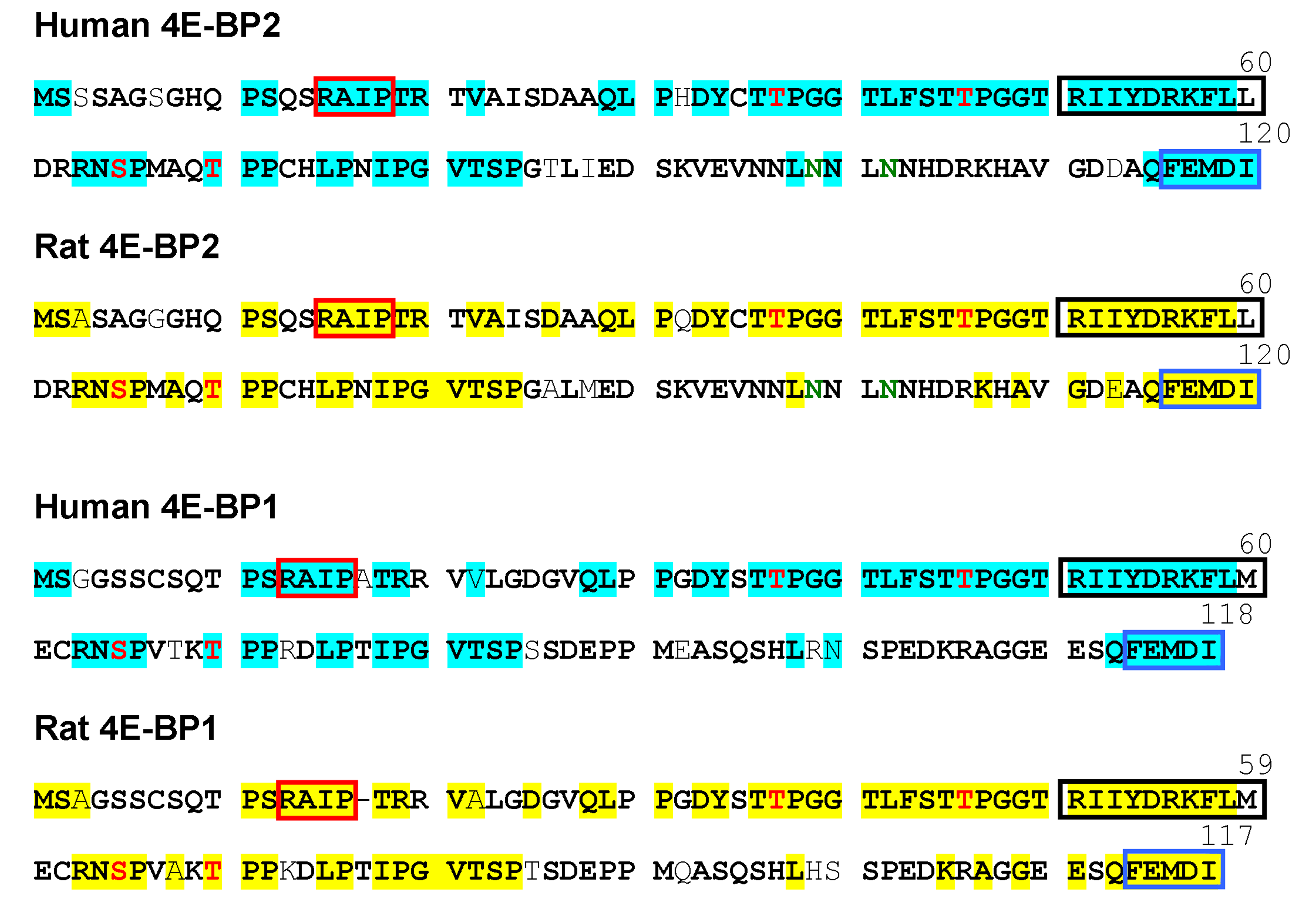

Supplement: S4 Fig — Identical amino acids between 4E-BP1 and 4E-BP2 are marked in blue and yellow in the human and rat sequence, respectively. Homology between human and rat is marked in bold type. The phosphorylation regulation sites are marked in red; the amino acids susceptible to deamidation in green. The eIF4E binding site [24] is boxed in black; the TOS motif [25] in blue; and the RAIP sequence [26] in red. Sequences were obtained from UniProtKB database (http://www.uniprot.org/). (TIF) [file pone.0121958.s004.tif]
